# Potential role of the N-MYC downstream-regulated gene family in reprogramming cancer metabolism under hypoxia

**DOI:** 10.18632/oncotarget.10684

**Published:** 2016-07-18

**Authors:** Ga Young Lee, Yang-Sook Chun, Hyun-Woo Shin, Jong-Wan Park

**Affiliations:** ^1^ Department of Biomedical Sciences, Seoul National University College of Medicine, Seoul, Republic of Korea; ^2^ Department of Pharmacology, Seoul National University College of Medicine, Seoul, Republic of Korea; ^3^ Ischemic/Hypoxic Disease Institute and Cancer Research Institute, Seoul National University College of Medicine, Seoul, Republic of Korea

**Keywords:** cancer, metabolic reprogramming, hypoxia, NDRG

## Abstract

Metabolic reprogramming toward aerobic glycolysis and lactate fermentation supplies cancer cells with intermediate metabolites, which are used as macromolecule precursors. The oncogene *MYC* contributes to such aerobic metabolism by activating the expression of numerous genes essential for glycolysis and mitochondrial biogenesis. However, to survive and evolve in a hypoxic tumor milieu, cancer cells must revise MYC-driven metabolism because the mitochondrial respiratory chain provides free electrons to generate oxygen free radicals with inefficient production of ATP due to oxygen depletion. Instead, hypoxia-inducible transcription factor hypoxia-inducible factor 1 (HIF-1) takes over the role of MYC in glycolysis, but suppresses mitochondrial biogenesis and activity to protect cells from such threats. Recently, the N-MYC downstream-regulated gene (NDRG) family has received attention as potential biomarkers of cancer prognosis. NDRGs are repressed MYC-dependently in various cancers, but induced under hypoxia because HIF-1 directly activates their promoters and indirectly de-represses them by antagonizing MYC. In this review, we summarize the current understanding of the reprogramming of cancer metabolism via the counterbalance between MYC and HIF-1, and discuss the proven and putative roles of the NDRG family in adjusting cancer metabolism according to the ambient oxygen level.

## INTRODUCTION

Cellular metabolism transition is a hallmark of cancer [1]. Positron-emission topography (PET), an imaging technique that uses the radiolabeled tracer 2-[18F]-fluoro-2-deoxy-D-glucose (FDG), enables visualization of glucose utilization in cancer patients and has shown that an increased rate of glycolysis is almost universally present in primary and metastatic tumors [2]. The augmentation of glycolysis and lactate fermentation in cancer cells even under aerobic conditions was first discovered 60 years ago by Otto Warburg, and is now termed the Warburg effect [3].

The Warburg effect is associated with a paradox; that is, why aerobic glycolysis that yields only 4 ATPs per glucose would be preferred over oxidative phosphorylation that generates up to 36 ATPs per glucose [4]. At that time, Otto Warburg made an erroneous postulation that the seemingly “less efficient” aerobic glycolysis was due to a disturbance in mitochondrial respiration in cancer cells [5, 6]. However, it is now established that cancer cells must be continuously supplied with ‘building blocks’, such as nucleotides, amino acids, and lipids, to achieve replicative immortality. In particular, an adequate amount of glucose metabolites must be available as macromolecule precursors, rather than for ATP generation [7]. Hence, cancer metabolism appears to be adapted to the anabolic program, which is under direct management by various oncogenes, such as *MYC* and hypoxia-inducible factor 1 (*HIF-1*) [8, 9]. The glycolytic phenotype of cancer cells has been suggested to be an adaptive response to hypoxia, which not only supports proliferation but also alters the surrounding microenvironment by overwhelming neighboring normal cells due to increased acid production [10].

We begin by exploring the partnership between MYC and HIF-1 in reprogramming cancer metabolism. We then introduce N-MYC downstream-regulated gene (NDRG) family members as novel oxygen-dependently regulated genes that can manipulate MYC-HIF tumor metabolic pathways and ultimately modify the Warburg effect.

## MYC AND HIF-1: MASTER REGULATORS OF METABOLIC REPROGRAMMING

### MYC regulates normoxic energy metabolism

Aside from the expression of its target genes essential for cell cycle progression, the oncogene *MYC* stimulates cancer growth by re-engineering the metabolic system. MYC facilitates cellular glucose uptake by inducing the expression of glucose transporter 1 (GLUT1), and activates the Warburg effect by inducing production of a number of glycolytic enzymes, including hexokinase (HK2), phosphofructokinase (PFKM), enolase 1 (ENO1), and pyruvate kinase (PKM2). Furthermore, MYC promotes mitochondrial biogenesis by activating the expression of mitochondrial transcription factor A (TFAM) and the enzymes necessary for mitochondrial metabolism [11, 12]. Overall, mitochondrial respiration and anabolic biosynthesis are promoted by MYC, allowing cancer cells to proliferate in oxygen- and nutrient-sufficient conditions. Conversely, the excessive activation of MYC can be cytotoxic because it drives cell death through p53-dependent and p53-independent pathways [13].

### HIF-1 governs hypoxic energy metabolism

HIF-1 is a heterodimeric transcription factor composed of HIF-1α and aryl hydrocarbon receptor nuclear translocator (ARNT), and its expression reversely correlates with oxygen availability. Under normoxia, HIF-1α is hydroxylated by the HIF prolyl-hydroxylase domain enzymes (PHDs), poly-ubiquitinated by the von Hippel Lindau (VHL)/E3 complex, and degraded through the 26S proteasome. Under hypoxia, however, HIF-1α escapes the hydroxylation and degradation processes, dimerizes with ARNT in the nucleus, and transactivates numerous genes for hypoxic adaptation [14, 15].

Like MYC, HIF-1 is responsible for the shift from oxidative to glycolytic metabolism by upregulating glucose transporters and various glycolytic enzymes. In contrast to MYC, HIF-1 strongly inhibits mitochondrial respiration and biogenesis. For instance, HIF-1 blocks entry of acetyl coenzyme A to the Krebs cycle by inducing pyruvate dehydrogenase kinase 1 (PDK1), which inactivates pyruvate dehydrogenase [16]. PDK1 not only suppresses the mitochondrial oxygen consumption rate but also decreases reactive oxygen species (ROS) production [17]. Furthermore, HIF-1 reduces the overall mitochondrial mass by inducing BCL2/Adenovirus E1B 19 kDa interacting protein 3 (BNIP3), which triggers mitochondrial degradation through autophagy and halts the excessive production of mitochondrial ROS [18]. Such metabolic adjustments may reduce energy-consuming anabolic synthesis, and increase cell survival during hypoxia [11, 19].

## THE NDRG FAMILY: MODULATORS OF METABOLIC REPROGRAMMING

### Structures and functions of NDRG proteins

**Figure 1:**
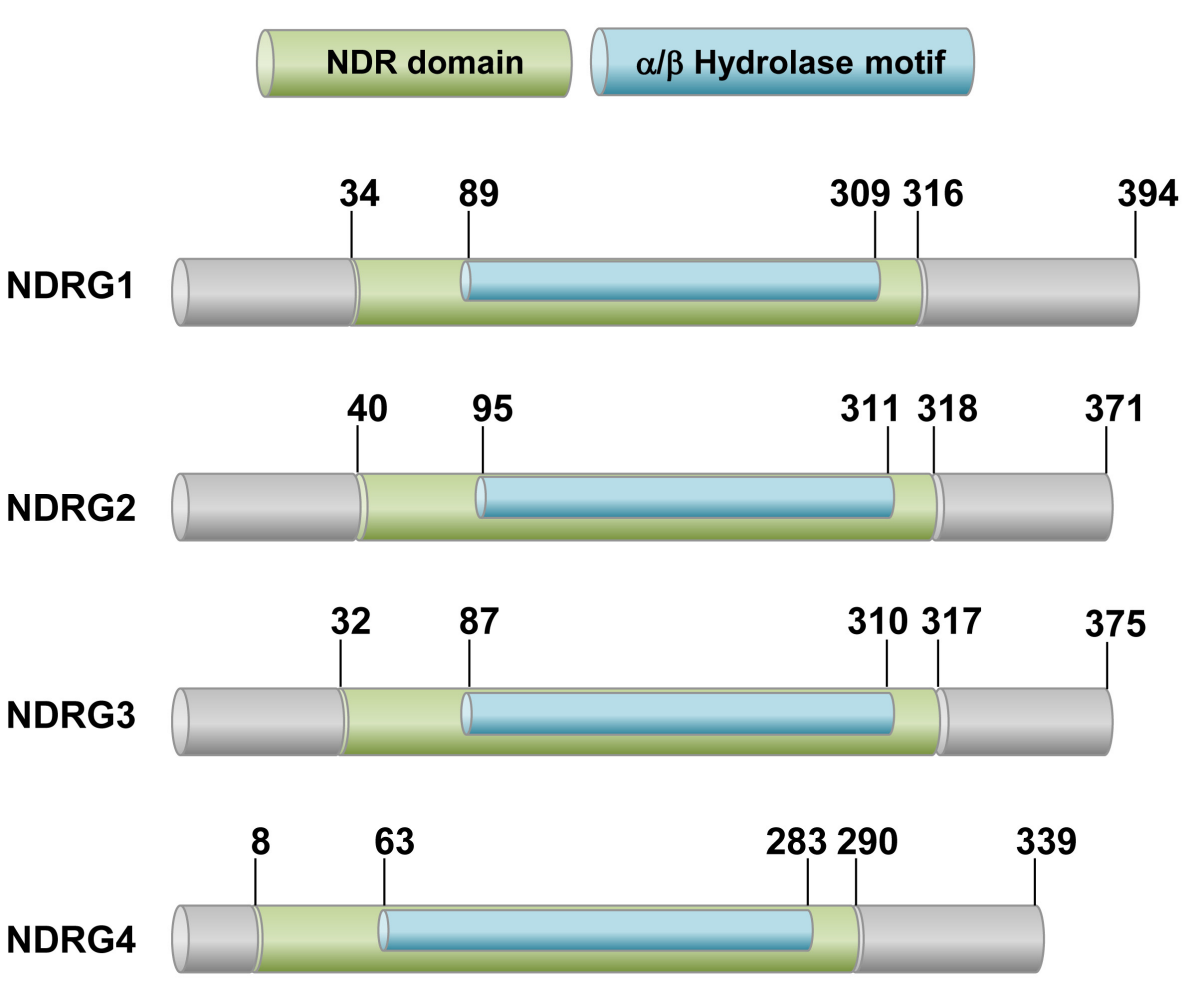
A schematic drawing of human NDRG proteins The NDRG family members are 53-65% homologous to each other and share the conserved NDR domain, which includes the alpha/beta hydrolase motif. However, they have no hydrolase activity due to lack of amino acid residues critical for catalytic reaction.

The human NDRG family consists of four members (NDRG1-4), which exhibit 53-65% homology. The NDRG1-4 genes encode polypeptides of 394, 371, 375, and 339 amino acids, respectively. Considering that NDRGs commonly share an α/β hydrolase fold region, they could be categorized into the α/β hydrolase superfamily (Figure 1) [20]. However, in contrast to typical α/β hydrolase superfamily members, NDRG proteins contain critical aberrations in their motifs. While a “nucleophile-acid-histidine” catalytic triad is present in the α/β hydrolase fold of typical α/β hydrolases, two residues in the triad are substituted by glycines in NDRGs. In addition, the conserved histidine in the fifth motif is absent in NDRGs. NDRG proteins have no hydrolase activity due to the absence of these critical residues [21, 22].

The tissue distribution of NDRGs varies. While NDRG1 is ubiquitously expressed, NDRG2 is expressed predominantly in the brain, heart, and skeletal muscle [23, 24]. NDRG3 is expressed primarily in the prostate and testes; NDRG4 is expressed almost exclusively in the brain and heart [24, 25]. The *NDRG* genes are highly conserved across various species, alluding to their involvement in critical cellular functions [23]. *NDRG1*, which is alternatively named Cap43, Drg1 or Rit42, is the most intensively studied member and has been identified as a stress-responsive gene or a differentiation-related gene [26, 27]. NDRG1 might also be involved in maintenance of the myelin sheath of peripheral nerves [28]. However, the functions of the other NDRGs remain largely unexplored to date.

### Roles of NDRG proteins in cancer development

Guan *et al*. first reported the intimate connection between NDRG and cancer. They found that NDRG1 is downregulated in colon cancer and that NDRG1 overexpression inhibits cell migration *in vitro* and cancer metastasis *in vivo* [26]. Since then, the focus of studies on NDRG has shifted towards the involvement of NDRG in cancer progression, to the extent that the potential of NDRG as a prognostic biomarker is gaining attention. Table 1 summarizes the clinically evaluated roles of NDRGs in cancer progression. Despite the evidence for a strong association between NDRGs and cancer progression, the involvement of NDRGs in metabolic reprogramming has not been investigated extensively to date. Given the reports of the roles of NDRGs in the MYC and HIF signaling pathways, we here suggest a possible connection between NDRG and cancer metabolism, and predict the potential of NDRG for application in the ongoing battle against cancer.

**Table 1:** Clinical data-based summary of NDRG expression and function in human cancers

Name	Function	Cancer type	Expression	References
NDRG1	Tumor Suppressive	Breast ca.	Down in cancer ∝ good prognosis	[64] [63]
		Colorectal ca.		[71]
		Esophageal squamous cell ca.		[72]
		Glioma		[73]
		Neuroblastoma		[74]
		Oral and oropharyngeal squamous cell ca.		[75]
		Prostate ca.		[76]
		Pancreatic ca.		[77]
		Renal cell ca.		[78]
	Oncogenic	Cervical ca.	Up in cancer ∝ poor prognosis	[79]
		Colorectal ca.		[80]
NDRG2	Tumor Suppressive	Astrocytoma	Down in cancer ∝ good prognosis	[81]
		Colorectal ca.		[82-84]
		Esophageal ca.		[85]
		Gastric ca.		[86, 87]
		Glioma		[88]
		Hepatocellular ca.		[89]
		Meningioma		[90]
		Oral squamous ca.		[67]
		Pancreatic ca.		[91]
		Renal cell ca.		[92]
NDRG3	Oncogenic	Prostate ca.	Up in cancer ∝ poor prognosis	[24]
		Hepatocellular ca.		[48]
NDRG4	Tumor Suppressive	Colorectal ca.	Down in cancer ∝ good prognosis	[70]
		Glioblastoma		[93]

ca., cancer; Up, upregulated; Down, downregulated; ∝, “Its expression in cancer tissues correlates with”

### NDRGs are regulated through MYC and HIF-1 in an oxygen-dependent manner

MYC-dependent regulation of the NDRG family was first reported in 1999 by Shimono *et al*., who screened MYC target genes using *N-Myc*-mutated mouse embryos [29]. cDNA subtraction revealed a gene that was amplified 20-fold in the mutant embryos, which was thus designated *Ndrg1*. Since Ndrg1 expression was suppressed by co-expression of N-MYC and myc-associated factor X (MAX), the dimeric complex of N-MYC and MAX was suggested to repress the *Ndrg1* gene. As C-MYC also represses this gene, the MYC family members might share the ability to repress the Ndrg1 gene [29]. Downregulation of NDRG1 by both N-MYC and C-MYC was also reported by Li *et al*. [30]. Although the MYC-dependent regulation of other NDRG members was initially questionable [31], it was later demonstrated that MYC in association with MYC-interacting zinc finger protein (MIZ-1) does indeed transcriptionally repress NDRG2 [32]. NDRG2 was upregulated in intestinal and leukemia cells where MYC expression was repressed. By contrast, NDRG2 was downregulated in HeLa cells that overexpressed MYC under serum stimulation. As an MIZ-1 mutant antagonized the repression of NDRG2 by MYC, the MYC and MIZ-1 dimeric complex is likely involved in NDRG2 repression [32]. On the other hand, whether or not MYC also represses the *NDRG3* and *NDRG4* genes has not been clearly demonstrated so far.

Ambient oxygen tension plays a critical role in the regulation of NDRG expression. Hypoxia and hypoxia-mimicking compounds, such as nickel, cobalt, and iron chelators, increase NDRG1 expression at the transcriptional level, and NDRG1 has been suggested to be an effector molecule required for hypoxic adaptation [33-36]. NDRG2 expression is also increased under hypoxia and by nickel or cobalt ions [37]. Regulation of NDRG3 through hypoxic accumulation of lactate will be discussed in detail in the next section. Although the mechanism of NDRG4 regulation has not been fully investigated yet, a recent study reported that NDRG4 expression is elevated in rat cardiomyocytes subjected to hypoxia and re-oxygenation [38]. This suggests the possibility that NDRG4 is also induced in an oxygen-dependent fashion. However, as NDRG4 was induced at the transcriptional level through the TNF-α/NF-κB signaling pathway in the study, NDRG4 expression seems not to be regulated directly by hypoxia *per se*.

Mechanistically, the hypoxia-induced expression of NDRGs is associated with HIF-1. The presence of a hypoxia-response element (HRE) in the *NDRG1* and *NDRG2* genes and the HIF-1-dependent activation of the genes have been demonstrated in several studies. The NDRG1 promoter region between −1,202 to −450 base pairs harbors the conserved sequence for HIF-1 binding [39], and NDRG2 also contains an HRE site in its promoter [37]. This indicates that NDRG1 is constitutively upregulated in renal cancer cells which highly express HIF-1/2α due to deletion of the *VHL* gene [40, 41].

Aside from the direct activation of *NDRG* genes, HIF-1α is also expected to induce NDRG expression in an indirect manner. MYC and its upstream β-catenin are key oncogenic players that maintain the cell cycle under tumor-favorable conditions [42, 43]. Upon exposure to hypoxic conditions, however, cells must deactivate MYC and β-catenin to halt cell division, which is driven by HIF-1α. HIF-1α antagonizes MYC and β-catenin by dissociating their dimeric structures through a physical interaction [44-46]. In addition, HIF-1α induces the deacetylation of β-catenin by dissociating arrest defective 1 (ARD1; alternatively named NAA10) from β-catenin, resulting in functional repression of the β-catenin-TCF4 (T-cell factor 4) complex [47]. As the β-catenin/TCF4 complex is the transcriptional activator for the *MYC* gene, HIF-1α-mediated inhibition of β-catenin results in subsequent loss of MYC. Considering the critical contribution of MYC to NDRG repression, it is plausible that HIF-1α induces NDRG expression indirectly by counteracting the effect of MYC on NDRG repression. Therefore, MYC and HIF-1 are the main factors that balance the cellular NDRG levels depending on the oxygen tension.

### NDRG3 mimics the role HIF-1α in oxygen-dependent regulation

Unlike other NDRG members, which display tumor-suppressive properties, NDRG3 is believed to be oncogenic. Dr. Yeom and colleagues recently discovered that NDRG3 is induced under hypoxia through a novel mechanism [48]. Similar to the mechanism underlying oxygen-dependent degradation of HIF-1α, the NDRG3 protein is also hydroxylated under normoxia by the HIF prolyl-hydroxylase PHD2, ubiquitinated by the pVHL-E3 ligase complex, and finally destroyed through the 26S proteasome. Under hypoxia, however, lactate physically associates with NDRG3 and interferes with the pVHL targeting to NDRG3, thereby stabilizing NDRG3. Functionally, NDRG3 promotes tumor responses to hypoxia, such as anti-apoptotic, angiogenic, and proliferative processes, by activating the RAF (v-raf-1 murine leukemia viral oncogene homolog)-ERK (extracellular-signal-regulated kinase) pathway. Although NDRG3 is not directly regulated by HIF-1α, its induction is indirectly affected by HIF-1α because HIF-1α supplies the lactate required for NDRG3 stabilization by accelerating the glycolytic flow. Therefore, NDRG3 as well as HIF-1α may be targets controlling the aggressive behaviors of hypoxic cancer cells. Indeed, tumor growth in mice was successfully abolished by knocking-down HIF-1α and NDRG3 in combination [48]. This study accentuates the significance of metabolic reprogramming in carcinogenesis, since the strong hypoxic responses induced by NDRG3 depend on lactate which accumulates as an end-product of hypoxia-adjusted metabolism. It is also interesting that the lactate-dependent expression of NDRG3 mirrors that of HIF-1α, as HIF-1α has also been reported to be upregulated by lactate and pyruvate [49].

### NDRGs support HIF-mediated reprogramming of energy metabolism

Among the NDRG family, NDRG2 was first identified to participate in metabolic reprogramming. NDRG2 interacts with and degrades GLUT1 through the proteasome, thereby decreasing glucose uptake in breast cancer cells [50]. More recently, NDRG2 was found to induce a series of events that repress aerobic glycolysis. NDRG2 inhibits the expression of the *c-myc* gene by reducing the β-catenin levels in the cytoplasm and the nucleus. Consequently, MYC-dependent glycolytic genes, such as *glut1*, *hk2*, *pkm2*, and *ldh1*, and MYC-dependent glutaminolytic genes, such as *asct2* and *gls1*, are repressed by NDRG2. Thus, the tumor-suppressive action of NDRG2 may be attributed to its ability to compromise MYC-driven metabolic reprogramming in cancer cells [51].

The involvement of the highly conserved Wnt/β-catenin signaling cascade has been investigated extensively in carcinogenesis [52]. Recently, this pathway has begun to be a focus of research as an operator of cancer metabolic programming [53, 54]. The glycolytic enzymes PDK1 and monocarboxylate transporter 1 (MCT1) were identified as target genes of β-catenin, revealing the existence of the Wnt-driven Warburg effect. Moreover, when the Wnt/β-catenin pathway was blocked using a TCF mutant, the growth of colon cancer was attenuated [55]. Indeed, since MYC is a well-known downstream target gene of Wnt/β-catenin [56, 57], blocking Wnt/β-catenin leads to MYC suppression. NDRG1 is reported to deregulate the Wnt singaling pathway. NDRG1 interacts with the Wnt coreceptor LRP6 (LDL receptor-related protein 6) and inhibits its phosphorylation at Ser1490, blocking the LRP6 activation by Wnt ligands. Such an effect of NDRG1 results in significant suppression of the epithelial-to-mesenchymal transition (EMT) [58]. Although this study focused on metastasis, we anticipate that LRP6 inhibition could underlie the effect of NDRG1 against Warburg metabolism.

The PI3K-Akt-mTOR (mammalian target of rapamycin) axis is another major regulatory pathway that promotes the Warburg effect and anabolic synthesis [59]. Following activation of this pathway by external growth factor signaling, glucose uptake is increased due to stimulation of GLUTs (1, 2 and 4) and upregulation of key glycolytic enzymes such as HK2 and PFKM1 [60]. Moreover, lipid biosynthesis is increased, which promotes de novo syntheses of lipogenic proteins by activating mTOR [61]. NDRG1 upregulates PTEN (phosphatase and tensin homolog deleted on chromosome 10), which is a key negative regulator of the PI3K pathway, and by doing so inactivates mTOR-mediated synthesis of proteins in pancreatic cancer [62]. Similarly in prostate cancer, NDRG1 overexpression was shown to increase PTEN expression and reduce the cellular level of phospho-Akt, while silencing of NDRG1 achieved the opposite effect [63]. Interestingly, NDRG1 expression is enhanced by PTEN at the transcriptional level [64], and such a positive feedback loop further boosts NDRG1-driven suppression of the PI3K-Akt pathway. From a functional perspective, PTEN is deactivated by phosphorylation at the Ser380/Thr382/Thr383 residues in the carboxyl-terminal region [65]. NDRG2 is capable of activating PTEN by dephosphorylating the serine-threonine cluster, and loss of NDRG2 decreases the functionality of PTEN and subsequently activates the PI3K-Akt pathway in adult T-cell leukemia-lymphoma [66]. In squamous cell carcinoma, ectopic expression of NDRG2 reversed aberrant activation of the PI3K-Akt pathway [67]. Likewise, NDRG2-overexpressing breast cancer cells exhibited a reduced level of p-Akt even after stimulation with IGF-1 (Insulin-like growth factor 1) [68]. Therefore, NDRG2 is regarded as a tumor-suppressor gene that represses the PI3K-Akt pathway [69]. NDRG4 has also been reported to induce the dephosphorylation of Akt in colorectal cancer [70].

In summary, NDRG family members directly or indirectly participate in metabolic reprogramming under hypoxia. NDRG1 inhibits the Wnt/β-catenin pathway by blocking LRP6 activation, which is expected to suppress the MYC-driven metabolic reprogramming. NDRG1, −2 and −4 inhibit the PI3K-Akt-mTOR pathway that stimulates the Warburg metabolism, and NDRG1 and −2 boost the inactivation of this pathway by stimulating PTEN, a bona fide inhibitor of the PI3K signaling. However, the involvement of NDRG3 in metabolic reprogramming has not been reported so far. As NDRG3 is upregulated under oxygen-deficient and lactate-enriched conditions, it is assumed that NDRG3 may play a role in metabolic adjustment to hypoxia, but such a possibility remains an open question.

**Figure 2:**
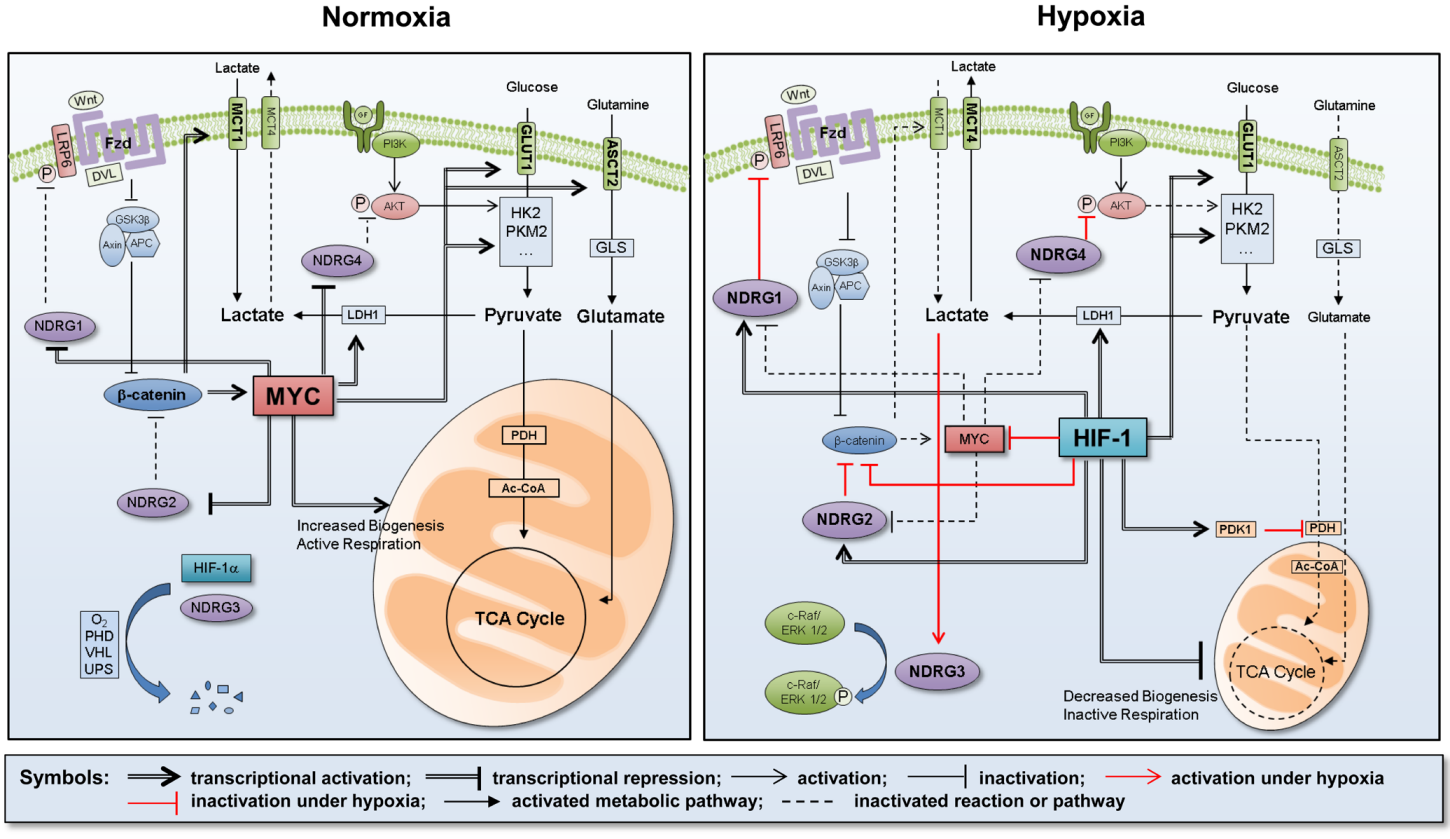
Summary of metabolic reprogramming under hypoxia Under normoxia, the energy metabolism is mainly govern by MYC, which activates the glycolytic flow by transcriptionally expressing a series of glycolytic enzymes, such as GLUT1, HK2, PKM2, and LDH1. Since NDRG members are normally repressed by MYC, the mitochondrial energy metabolism is maintained as an active state. Under hypoxia, however, the energy metabolism is govern by HIF-1 instead of MYC. HIF-1 keeps the glycolytic flow by expressing glycolytic enzymes but restricts the mitochondrial energy metabolism, which is attributed to reduced biogenesis of mitochondria and to increased PDK1, an inhibitor of pyruvate dehydrogenase. HIF-1 also upregulates NDRG1, −2 and −4 by directly activating these genes and by indirectly blocking the MYC-mediated repression of these genes. NDRG1 inhibits the Wnt/β-catenin pathway by blocking LRP6 phosphorylation and activation. β-catenin is suppressed by both NDRG2 and HIF-1, leading to further repression of MYC. NDRG4 inhibits AKT phosphorylation and activation, thereby suppressing the PI3K-mediated glycolysis. NDRG3 is stabilized in hypoxia by lactate accumulation, and induces various hypoxic responses by activating the c-Raf-ERK pathway. Consequently, HIF-1 and NDRGs cooperatively achieve metabolic reprogramming under hypoxia.

## CONCLUSIONS

Metabolic reprogramming, which is critical for tumor progression, is currently considered an emerging target for cancer therapy. In this review, we summarized the critical roles of MYC and HIF-1 in adjusting cancer metabolism to the ambient oxygen level and discussed the proven and putative roles of the NDRG family in regulating cancer metabolic reprogramming. The roles of these critical factors in metabolic reprograming are summarized in Figure 2. We here emphasized that the NDRG family may be a safety switch that turns off tumor-favorable metabolism and may represent a new strategy in the long battle against cancer.
